# Repeated administration of alpha-galactosylceramide ameliorates experimental lupus nephritis in mice

**DOI:** 10.1038/s41598-018-26470-w

**Published:** 2018-05-29

**Authors:** Takahiro Uchida, Hiroyuki Nakashima, Akira Yamagata, Seigo Ito, Takuya Ishikiriyama, Masahiro Nakashima, Shuhji Seki, Hiroo Kumagai, Naoki Oshima

**Affiliations:** 1 Department of Nephrology and Endocrinology, National Defense Medical College, Tokorozawa, Saitama Japan; 2 Department of Immunology and Microbiology, National Defense Medical College, Tokorozawa, Saitama Japan

## Abstract

Lupus nephritis is a crucial complication of systemic lupus erythematosus. In this study, we investigated the roles of mouse natural killer T (NKT) cells in lupus nephritis. From 24 weeks of age, NZB/NZW F1 mice were injected with alpha-galactosylceramide (α-GalCer) or vehicle once a week for four weeks. In the α-GalCer group, the levels of proteinuria and blood urea nitrogen were significantly lower than those in the vehicle group. The histological evaluation showed a decrease in glomerular immune complex deposits and an alleviation of podocyte injury. The proportion of NKT cells in the mononuclear cell (MNC) fraction in the α-GalCer group was significantly decreased in the liver, kidney, and spleen. The proliferation and cytokine production in α-GalCer-stimulated liver MNCs were markedly diminished in the α-GalCer group (anergy). The IFN-γ production in liver MNCs stimulated by concanavalin A or an anti-CD3 antibody did not differ between the two groups, whereas the IL-4 production was significantly lower in the α-GalCer group. In addition, the IgM production in CpG-oligodeoxynucleotide-stimulated spleen MNCs was significantly lower in the α-GalCer group. These results suggest that α-GalCer suppressed Th2 immune responses in NKT cells and B cell function, thereby slowing the progression of lupus nephritis.

## Introduction

Systemic lupus erythematosus (SLE) is one of the representative systemic autoimmune diseases, which is characterized by the presence of autoantibodies and involves virtually any organ. Lupus nephritis represents a crucial complication because renal outcomes in patients with lupus nephritis are poor despite the use of immunosuppressive therapy^1^. A previous study has shown that approximately one-third of patients with lupus nephritis progress to end-stage renal disease within 20 years^2^. An understanding of its detailed pathogenesis is urgently needed to find better therapeutic approaches.

Mouse natural killer T (NKT) cells are innate immune cells that express both the NK1.1 antigen and the T cell receptor (TCR) and are abundant in the liver. Once activated by IL-12 or an anti-CD3 antibody (Ab), they produce cytokines and exert antitumor cytotoxicity^3,4^. Their TCRs are mainly encoded by the Vα14Jα18 and Vβ8 genes^5,6^. The gene arrangement for encoding the TCR is invariant, and therefore, they are also known as invariant NKT (iNKT) cells. The functions of NKT cells have been examined under stimulation with alpha-galactosylceramide (α-GalCer), a specific sphingoglycolipid ligand of these cells^7^.

Liver NKT cells activated by α-GalCer trigger a potent antitumor response mediated via NK cells and subsequently CD8^+^ T cells^8,9^. However, NKT cells themselves can cause multiple organ failure, especially in aged mice^8,10^. On the other hand, NKT cells may play protective roles in some glomerulonephritis or vasculitis models^11,12^. In a SLE model, the expansion of NKT cells is thought to be involved in the onset of lupus nephritis^13^, whereas their immunoregulatory roles were also reported both in human SLE and SLE models^14–16^.

The effects of α-GalCer in the progression of autoimmune disease models have also been controversial. For example, it was shown that α-GalCer prevented the onset of diabetes in non-obese diabetic (NOD) mice, a representative type 1 diabetes model^17,18^. Conversely, there have been contradictory reports regarding the effects of α-GalCer in NZB/NZW F1 (BWF1) mice, an experimental model of SLE in which lupus nephritis-like lesions develop. Thus, one report showed that α-GalCer-activated NKT cells exacerbated the experimental lupus nephritis^19^, whereas a long-term reduction in severe proteinuria following α-GalCer treatment was reported in another study^20^.

Therefore, in the present study, we have investigated the roles of NKT cells in lupus nephritis using BWF1 mice. We show that the repeated administration of α-GalCer into BWF1 mice induced beneficial effects, as follows: (i) it improved proteinuria which represents a hallmark of renal injury, by protecting nephrin, a key functional molecule in the slit diaphragm of the podocytes^21^; (ii) it suppressed B cell function and decreased glomerular immune complex deposits; (iii) it induced not only an anergic state to α-GalCer in NKT cells, but also decreased the number of NKT cells in multiple organs and the production of IL-4 by these cells.

## Results

### The effects of α-GalCer treatment on the progression of kidney dysfunction

**Figure 1:**
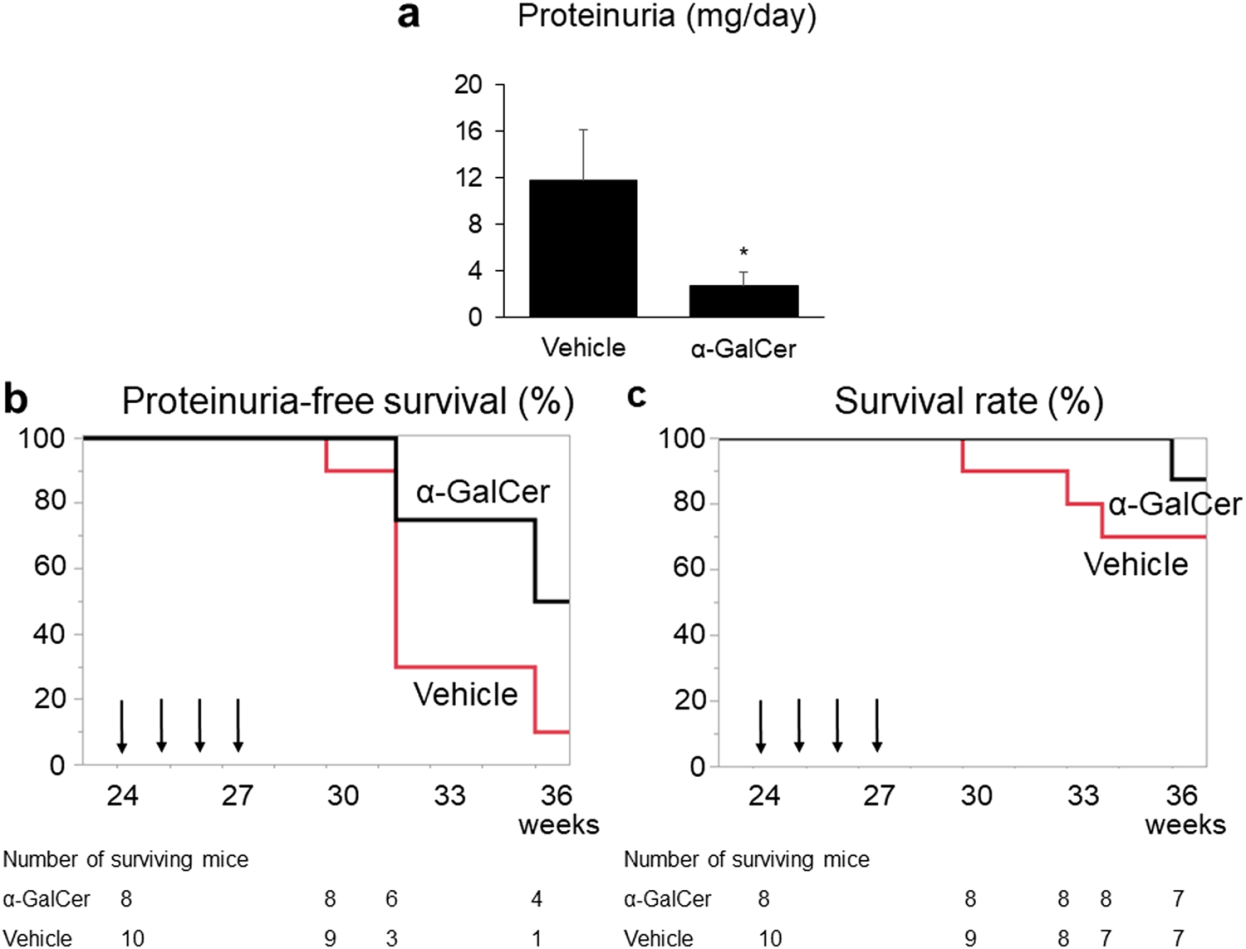
The effects of repeated alpha-galactosylceramide (α-GalCer) administration on proteinuria and survival rates. (**a**) The levels of proteinuria in each group, measured 8 weeks after the last α-GalCer or vehicle injection (*n* = 5–7 in each group). ^*^p < 0.05 compared with the vehicle group. (**b**) The percentages of mice with free of proteinuria and (**c**) the survival rate in the α-GalCer and the vehicle groups (*n* = 8 and 10, respectively). Arrows show the timing of when the mice were injected with α-GalCer or vehicle.

The levels of proteinuria in the α-GalCer group were significantly lower than those in the vehicle group (Fig. 1a). Moreover, the incidence of proteinuria in the α-GalCer group was also significantly lower when analyzed using the log-rank test (Fig. 1b, *p* < 0.05), but was not statistically significant when using the Cox proportional hazard model (Supplementary Table S1, *p* = 0.10). Neither group displayed hematuria. The survival rate in the α-GalCer group tended to be higher than that in the vehicle group, although it was not statistically different (Fig. 1c, *p* = 0.20 and Supplementary Table S1, *p* = 0.33).

The blood urea nitrogen (BUN) levels in the α-GalCer group were significantly lower than those in the vehicle group (Table 1). Moreover, the serum albumin levels in the α-GalCer group were significantly higher than those in the vehicle group, reflecting the difference in the levels of proteinuria between the two groups (Fig. 1a). Although no significant differences in the total serum protein levels or IgM levels were observed, the serum albumin/globulin ratio was significantly higher in the α-GalCer group. However, the serum IgG anti-dsDNA Ab levels did not differ between the two groups. The alanine aminotransferase levels were within the normal range and no significant difference was found between the two groups. Splenomegaly was observed in both groups; however, there was no difference in spleen weight or body weight. Furthermore, obvious lymphadenopathy was not observed in any of the mice.

**Table 1:** Biochemical and physiological parameters at the end of the experiment.

	Vehicle group	α-GalCer group
Number	5	7
Blood urea nitrogen (mg/dL)	41.0 ± 34.0	17.3 ± 5.6*
Serum albumin (g/dL)	1.84 ± 0.31	2.24 ± 0.21*
Total protein (g/dL)	5.86 ± 0.21	6.09 ± 0.47
IgM (mg/dL)	269 ± 44	200 ± 16
Albumin/globulin ratio	0.46 ± 0.11	0.58 ± 0.03*
Serum IgG anti-dsDNA Ab (U/mL)	1734 ± 1804	1805 ± 1256
Alanine aminotransferase (IU/L)	28.0 ± 3.6	24.6 ± 3.6
Spleen weight (g)	0.18 ± 0.05	0.22 ± 0.04
Body weight (g)	39.8 ± 1.0	38.9 ± 1.3

Data are presented as mean ± SEM or number.

^*^p < 0.05 (Student’s *t*-test).

α-GalCer: alpha-galactosylceramide.

### Glomerular immune complex deposits and the infiltration of inflammatory cells decrease following α-GalCer treatment

**Figure 2:**
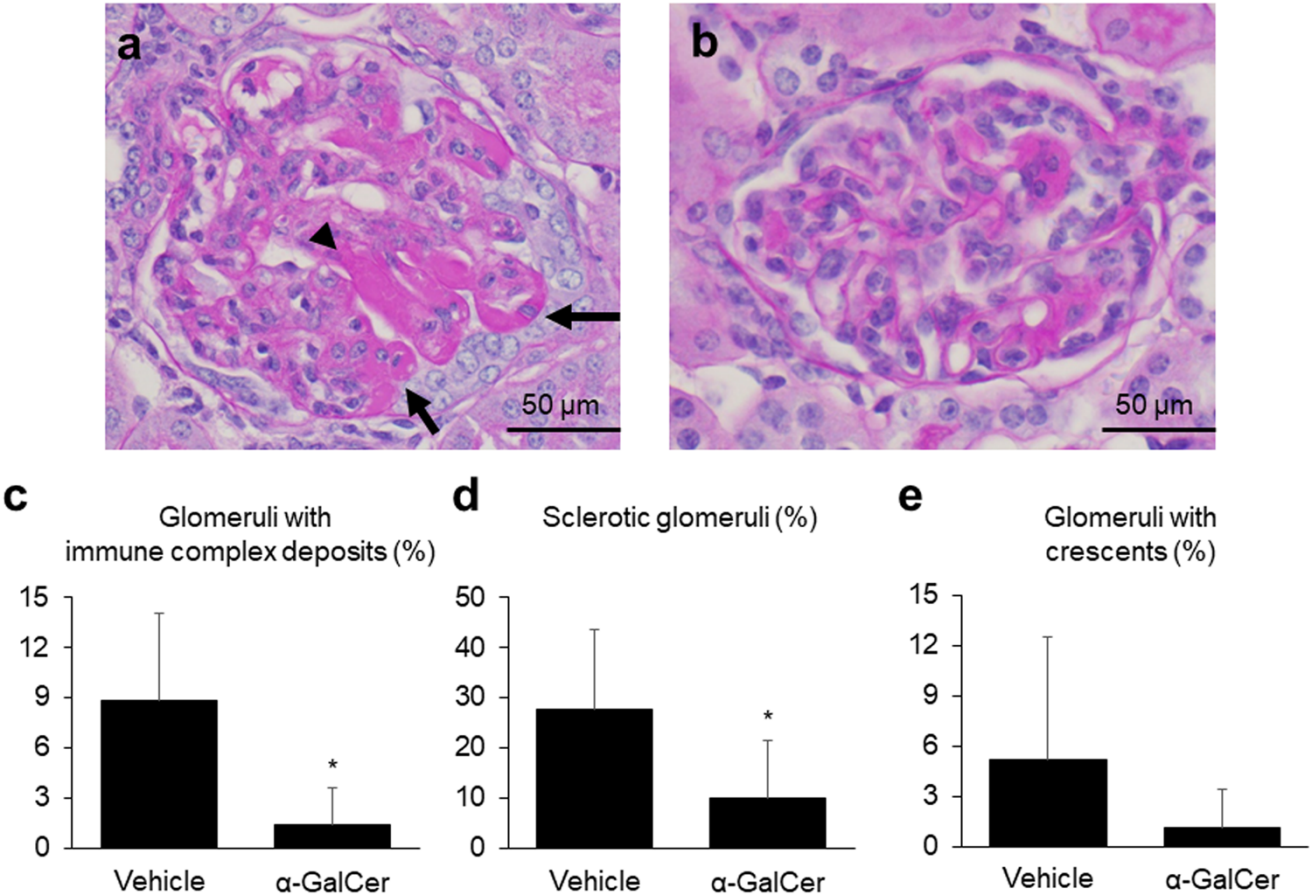
Kidney pathology of experimental lupus nephritis is ameliorated by treatment with alpha-galactosylceramide (α-GalCer). Representative images of periodic acid-Schiff staining of kidney slices in the (**a**) vehicle and (**b**) α-GalCer groups. Arrows, immune complex deposits; arrowhead, segmentally sclerotic area. The percentages of (**c**) glomeruli with immune complex deposits, (**d**) sclerotic glomeruli, and (**e**) glomeruli with crescents in each group (vehicle group; *n* = 5, α-GalCer group; *n* = 7). ^*^p < 0.05 compared with the vehicle group.

A representative image of periodic acid-Schiff (PAS) staining in the vehicle group is shown in Fig. 2a, while that in the α-GalCer group is shown in Fig. 2b. The percentage of glomeruli containing immune complex deposits and that of sclerotic glomeruli in the α-GalCer group were significantly lower (Fig. 2c,d). Furthermore, although the difference was not statistically significant, the percentage of glomeruli with crescents in the α-GalCer group tended to be lower than that in the vehicle group (Fig. 2e).

**Figure 3:**
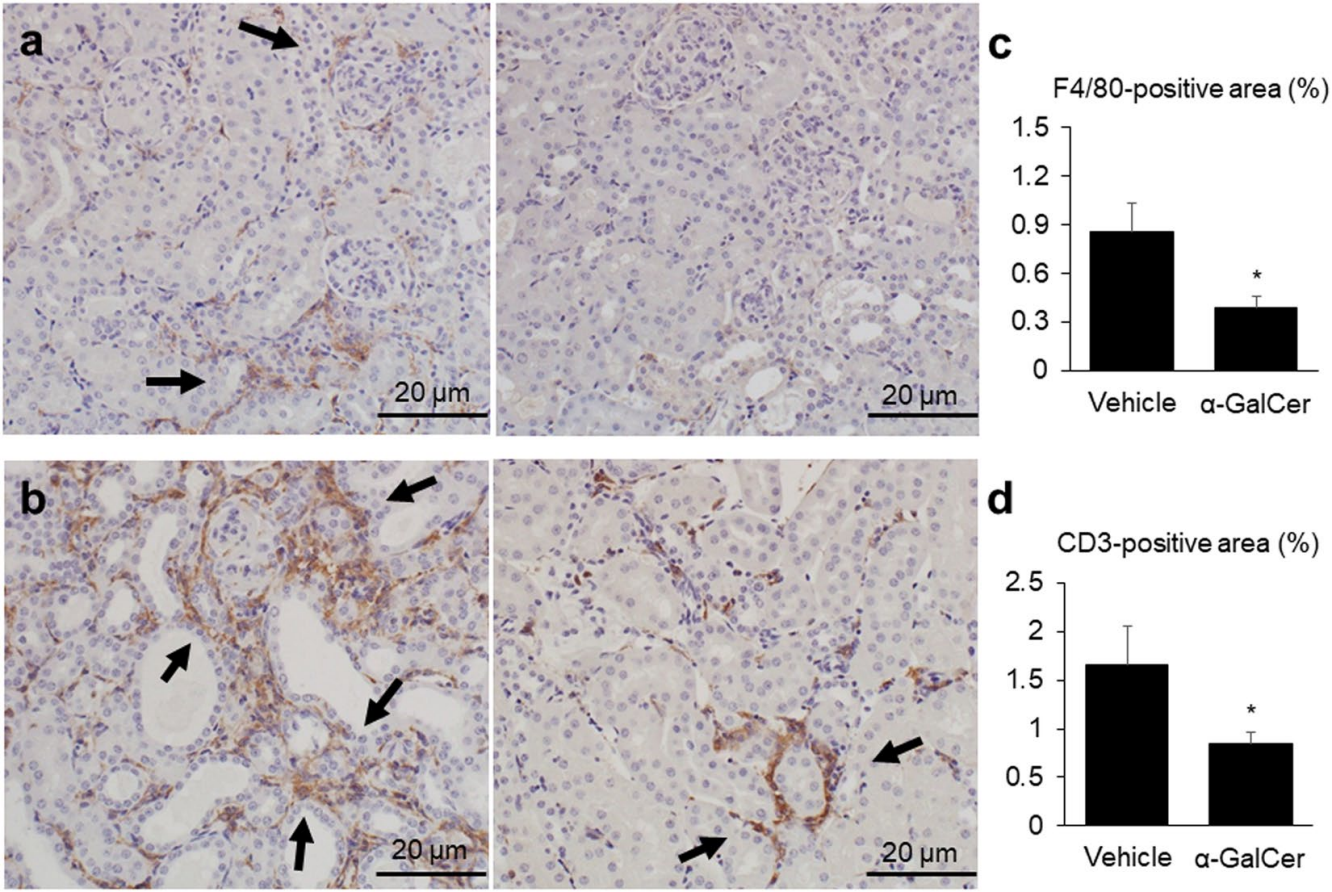
Alpha-galactosylceramide (α-GalCer) treatment decreases the infiltration of inflammatory cells into the kidney. Representative images of (**a**) F4/80-stained and (**b**) CD3-stained kidney slices in the vehicle (left) and α-GalCer (right) groups. In each panel, arrows indicate F4/80 or CD3-positive areas. The percentages of the (**c**) F4/80-positive area and (**d**) CD3-positive area in each group (vehicle group; *n* = 5, α-GalCer group; *n* = 7). ^*^p < 0.05 compared with the vehicle group.

We also evaluated the infiltration of inflammatory cells in the kidney. Representative images for F4/80 and CD3 staining in both groups are shown in Fig. 3a,b. Both the F4/80 and CD3 positive areas were significantly smaller in the α-GalCer group than those in the vehicle group (Fig. 3c,d). The renal messenger RNA (mRNA) expression levels for a dendritic cell marker (CD11c) and a plasma cell marker (CD138) did not significantly differ between the two groups (Supplementary Fig. S1).

### α-GalCer treatment decreases IgG deposition and ameliorates podocyte injury

**Figure 4:**
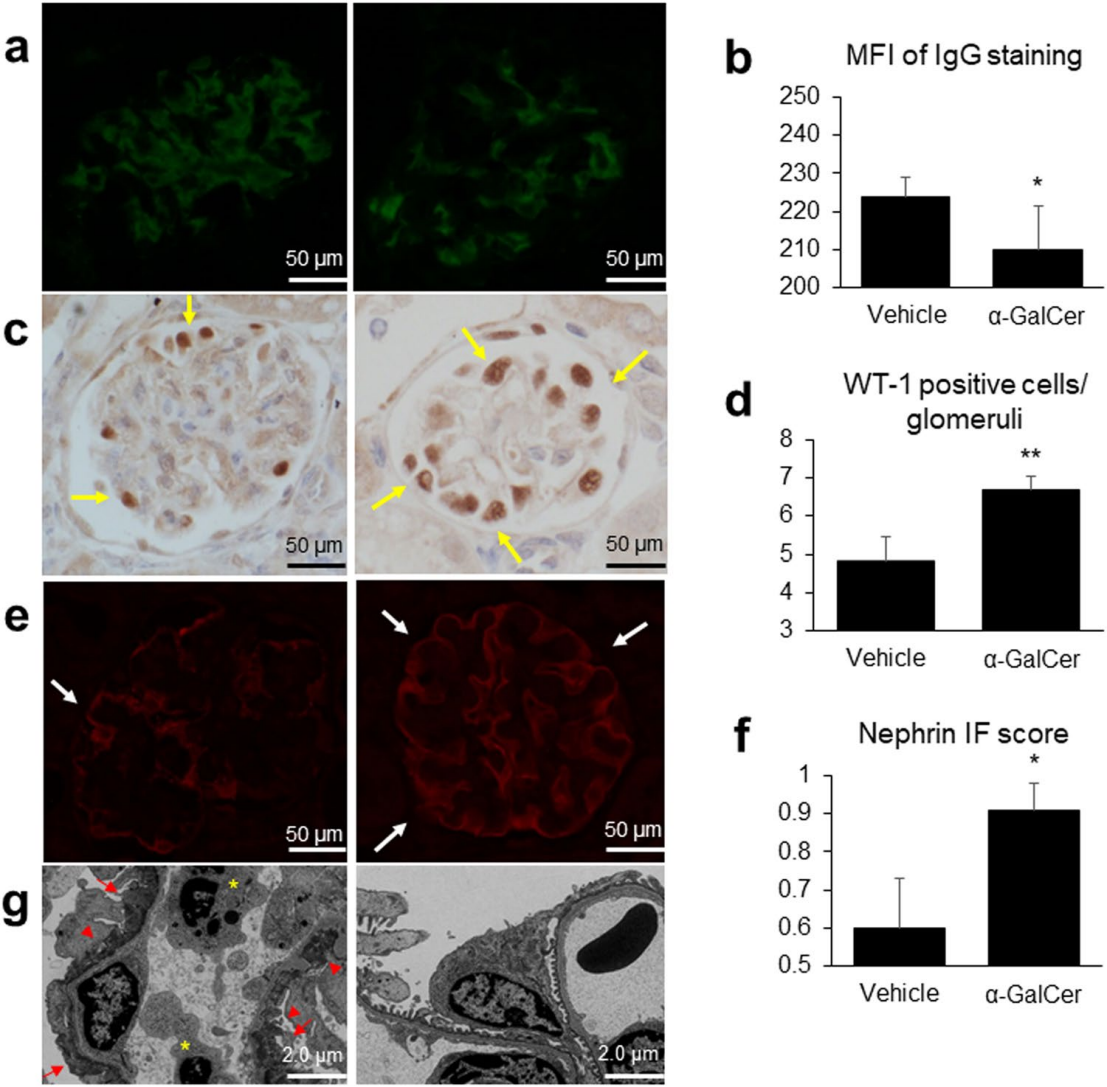
Alpha-galactosylceramide (α-GalCer) treatment decreases the deposition of glomerular immune complexes and ameliorates podocyte injury. Representative images of (**a**) immunofluorescence (IF) IgG staining, (**c**) immunoperoxidase WT-1 staining, and (**e**) IF nephrin staining of kidney slices in the vehicle (left) and α-GalCer (right) groups. In (**c**) and (**e**), arrows indicate positively stained cells or areas. (**b**) MFI (mean fluorescence intensity) of IgG staining, (**d**) WT-1 positive cells/glomeruli, and (**f**) semiquantitative nephrin IF score in each group (vehicle group; *n* = 5, α-GalCer group; *n* = 7). ^*^p < 0.05, ^**^p < 0.01 compared with the vehicle group. (**g**) Representative electron microscopy photomicrographs of kidney slices in the vehicle (left) and α-GalCer (right) groups. Widespread effacement of podocyte foot processes (red arrows) and electron dense immune deposits (red arrowheads) are shown in the vehicle group. Endocapillary infiltration of inflammatory cells was also observed (yellow asterisks).

Next, we assessed the deposition of glomerular immune complexes and the podocyte damage of the two groups. Figure 4a shows representative images of immunofluorescence (IF) IgG staining. In accordance with the light microscopy findings, the mean fluorescence intensity (MFI) of IgG staining in the α-GalCer group was significantly weaker (Fig. 4b).

Figure 4c shows representative images of WT-1 immunostaining, which represents a marker of podocyte damage^22^. Compared with that in the vehicle group, the number of WT-1 positive cells was significantly larger in the α-GalCer group (Fig. 4d). The IF nephrin staining score was also significantly higher in the α-GalCer group (Fig. 4e,f). These results suggested that podocyte injury was alleviated in the α-GalCer group.

Moreover, electron microscopy showed that although widespread effacement of podocyte foot processes was observed in the vehicle group, foot processes were relatively preserved in the α-GalCer group (Fig. 4g). Electron dense deposits, especially subepithelial deposits, were also smaller in the α-GalCer group.

### Repeated α-GalCer injection decreases the proportion of NKT cells and the cytokine responses to α-GalCer stimulation

**Figure 5:**
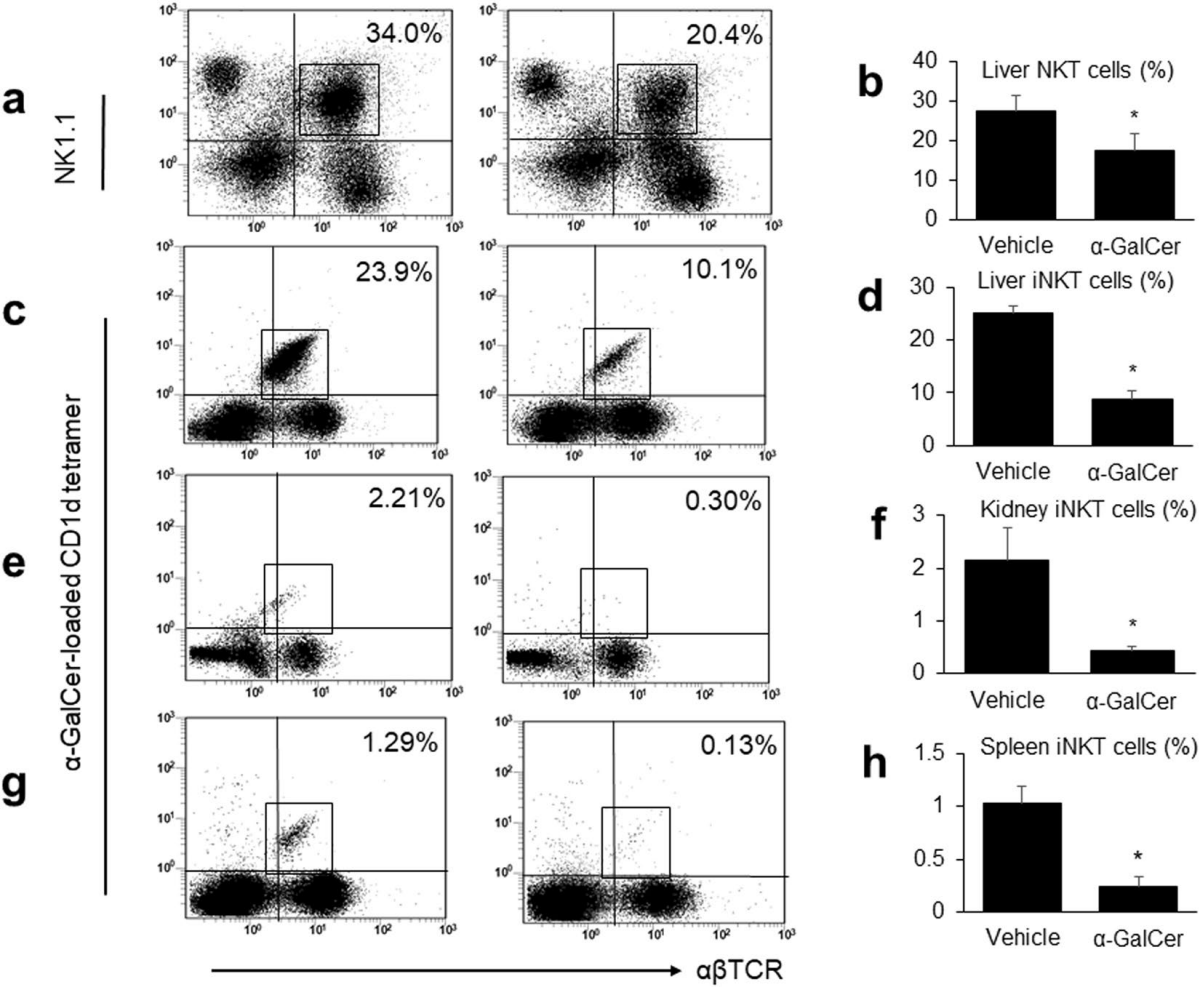
Repeated alpha-galactosylceramide (α-GalCer) administration decreases the proportion of NKT cells in multiple organs. (**a**) Flow cytometry analysis of liver mononuclear cells (MNCs) in the vehicle (left) and α-GalCer (right) groups, stained with anti-TCR αβ and anti-NK1.1 antibodies. In both panels, black squares show NKT cells. Flow cytometry analysis of (**c**) liver, (**e**) kidney, and (**g**) spleen MNCs in the (left) vehicle and (right) α-GalCer groups. Isolated MNCs were double stained with anti-TCR αβ antibody and α-GalCer-loaded CD1d tetramer; invariant NKT (iNKT) cells are shown in black squares. The percentages of (**b**) whole NKT cells in the liver and iNKT cells in the (**d**) liver, (**f**) kidney, and (**h**) spleen are shown (*n* = 4 in each group). ^*^p < 0.05 compared with the vehicle group.

We performed flow cytometry analysis to assess the number of NKT cells in multiple organs. Figure 5a shows representative images in which liver mononuclear cells (MNCs) were stained with anti-TCR αβ and anti-NK1.1 Abs. The proportion of NKT cells in the liver in the α-GalCer group was significantly reduced compared to that in the vehicle group (Fig. 5b). Figure 5c,e and g show representative images in which liver, kidney, and spleen MNCs were stained with anti-TCR αβ Ab and the α-GalCer-loaded CD1d tetramer. The proportions of iNKT cells in the liver, kidney, and spleen in the α-GalCer group were significantly lower than those in the vehicle group (Fig. 5d,f and h).

**Figure 6:**
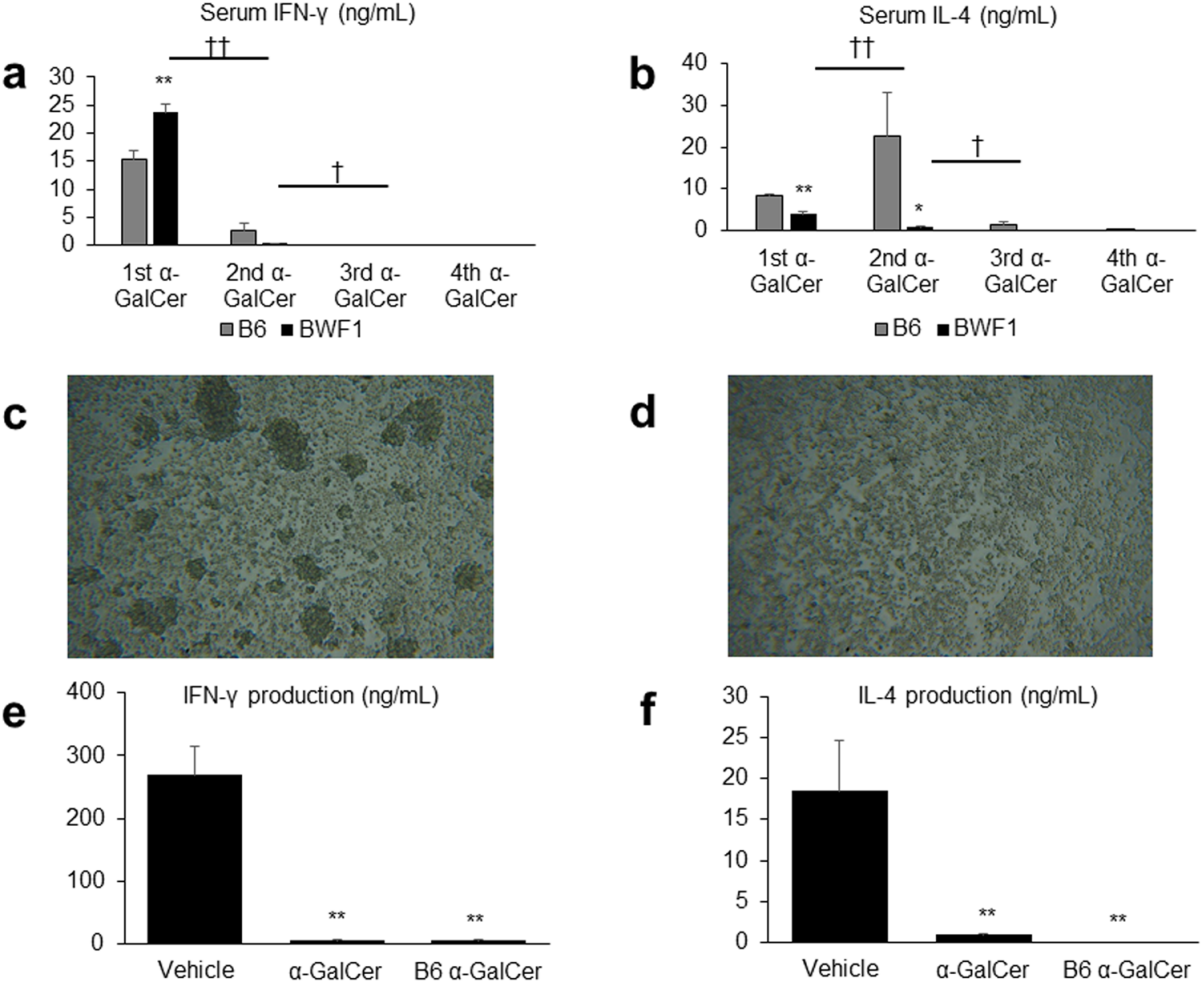
Repeated alpha-galactosylceramide (α-GalCer) injection induces anergy in NKT cells to α-GalCer. (**a**) Serum IFN-γ levels 12 hours after α-GalCer injection and (**b**) IL-4 levels 3 hours after α-GalCer injection in (NZB/NZW) F1 (BWF1) and C57BL/6 J (B6) mice. The mice were injected with 2 μg/body of α-GalCer once a week for 4 weeks, and cytokine levels were evaluated after every injection (*n* = 4 in each group). ^*^p < 0.05, ^**^p < 0.01 compared with B6 mice. ^††^*p* < 0.01 compared with the levels after the first α-GalCer injection. ^†^*p* < 0.05 compared with the levels after the second α-GalCer injection. Microphotographs of liver mononuclear cells (MNCs), stimulated by α-GalCer in the (**c**) vehicle and (**d**) α-GalCer groups (original magnification, 100×). *In vitro* production of (**e**) IFN-γ and (**f**) IL-4 from liver MNCs in BWF1 mice treated with α-GalCer or vehicle, and B6 mice treated with α-GalCer. Liver MNCs were obtained 8 weeks after the last α-GalCer or vehicle injection and were stimulated with α-GalCer for 48 hours (*n* = 4 in each group). ^**^p < 0.01 compared with the vehicle group of BWF1 mice.

Next, we assessed the cytokine responses under α-GalCer stimulation. The repeated administration of α-GalCer reduced the elevation of serum IFN-γ and IL-4 levels following α-GalCer injection in mice, and the levels of both cytokines decreased to undetectable levels (Fig. 6a,b). These results were also confirmed *in vitro*. Thus, α-GalCer-stimulated liver MNCs strongly proliferated in the vehicle group (Fig. 6c), whereas the proliferation was poor in the α-GalCer group (Fig. 6d). In addition, the IFN-γ and IL-4 levels produced by liver MNCs following α-GalCer stimulation were significantly lower in the α-GalCer group, even at 8 weeks after the last α-GalCer injection (Fig. 6e,f). Furthermore, not only BWF1, but also C57BL/6 J (B6) mice undergoing repeated α-GalCer administration showed similar cytokine responses both in *in vivo* and *in vitro* (Fig. 6a,b,e and f). These results suggested that the repeated administration of α-GalCer induced a state of anergy to α-GalCer, which was not specific to BWF1 mice.

### Repeated α-GalCer administration decreases B cell function through the suppression of Th2 immune responses

**Figure 7:**
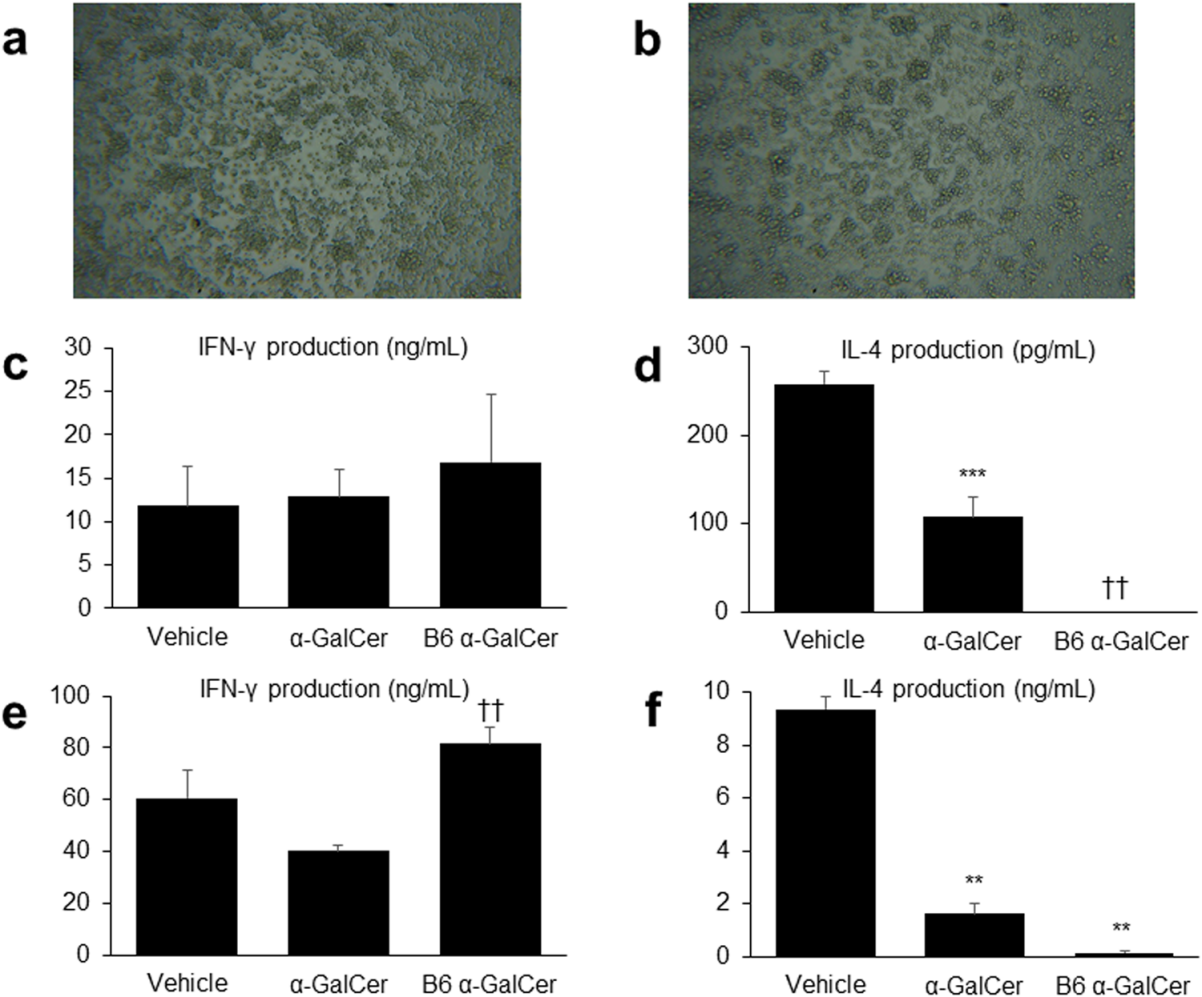
Th2 but not Th1 immune responses are suppressed by repeated alpha-galactosylceramide (α-GalCer) injections. Microphotographs of liver mononuclear cells (MNCs), stimulated by concanavalin A (ConA) in the (**a**) vehicle and (**b**) α-GalCer groups (original magnification, 100×). IFN-γ production and IL-4 production from liver MNCs, stimulated with (**c**,**d**) ConA or (**e**,**f**) anti-CD3 antibody for 48 hours in (NZB/NZW) F1 (BWF1) mice treated with α-GalCer or vehicle, and C57BL/6 J (B6) mice treated with α-GalCer (*n* = 4–5 in each group). ^**^p < 0.01, ^***^p < 0.001 compared with the vehicle group of BWF1 mice. ^††^*p* < 0.01 compared with the α-GalCer group of BWF1 mice.

The cytokine production levels of MNCs were also evaluated under stimulation with other agents. Liver MNCs stimulated with concanavalin A (ConA), a T-cell stimulant^23^, proliferated well both in the vehicle and α-GalCer groups (Fig. 7a,b), and the levels of IFN-γ produced by these cells were comparable between the two groups (Fig. 7c). However, the IL-4 levels in the α-GalCer group were significantly lower than those in the vehicle group (Fig. 7d). Similar cytokine responses were observed when liver MNCs were stimulated with an anti-CD3 Ab. Although the levels of IFN-γ produced by liver MNCs did not differ between the two groups, the IL-4 levels in the α-GalCer group were significantly lower than those in the vehicle group (Fig. 7e,f). This suppression of IL-4 production was also observed in B6 mice, following the repeated administration of α-GalCer (Fig. 7c–f).

**Figure 8:**
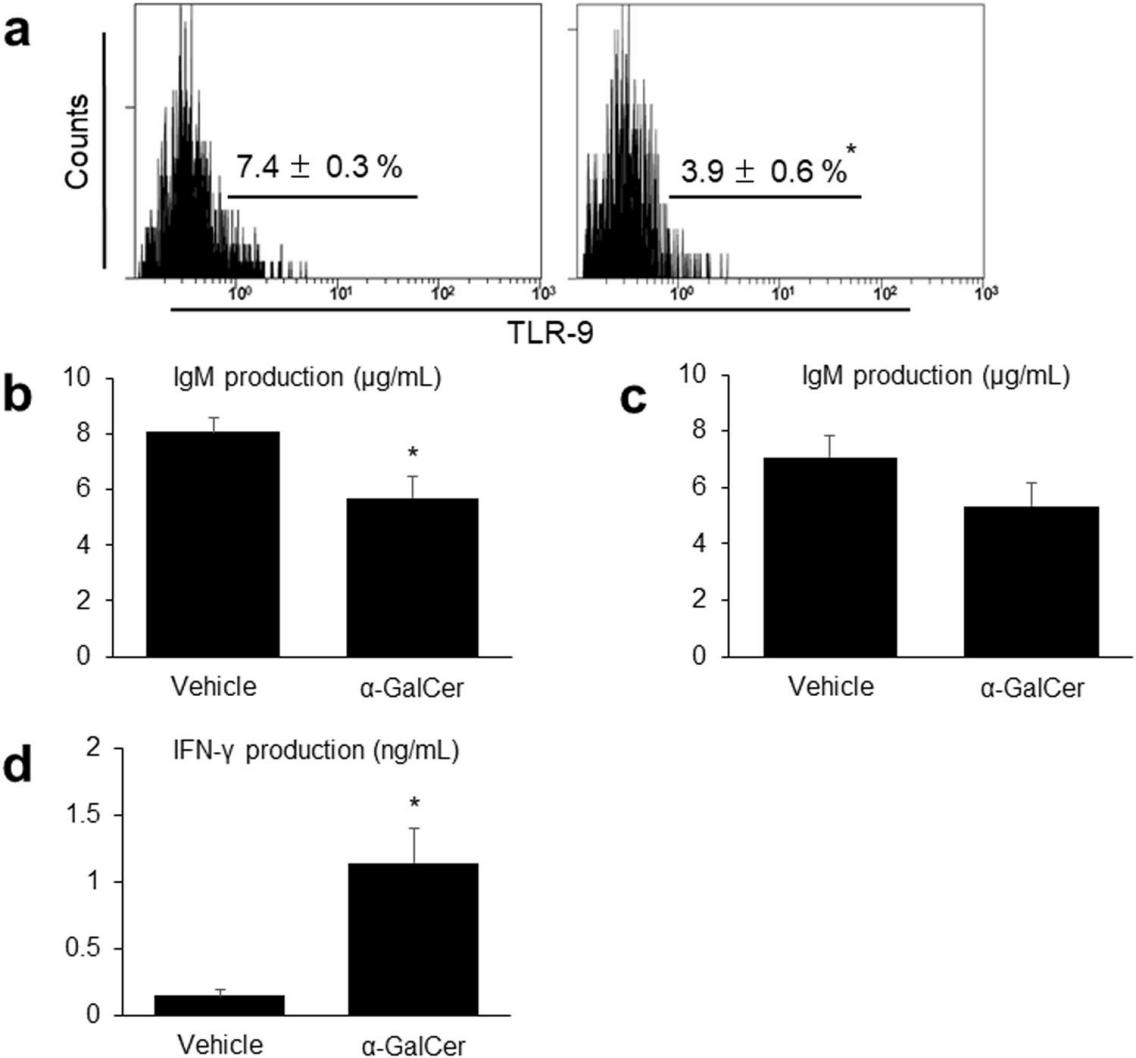
A decrease in B cell function and augmentation of NK cell function is observed in (NZB/NZW) F1 (BWF1) mice treated with alpha-galactosylceramide (α-GalCer). (**a**) Representative histograms obtained by flow cytometry showing the expression of intracellular Toll-like receptor-9 (TLR-9) in splenic F4/80^+^ CD11b^+^ macrophages in the vehicle (left) and α-GalCer (right) groups of BWF1 mice. The data represent the mean ± SEM in each group (*n* = 4 in each group). IgM production by spleen mononuclear cells (MNCs) stimulated with (**b**) CpG-oligodeoxynucleotide (CpG-ODN) or (**c**) lipopolysaccharide for 48 hours in each group (*n* = 4–6 in each group). (**d**) IFN-γ production by liver MNCs stimulated by CpG-ODN for 48 hours in each group (*n* = 4 in each group). ^*^p < 0.05 compared with the vehicle group.

To assess the B cell function, we measured the IgM production levels of splenic MNCs. The number of B cells was also evaluated by flow cytometry. In the α-GalCer group, the expression of Toll-like receptor-9 (TLR-9) which recognizes the CpG-oligodeoxynucleotide (CpG-ODN), common bacterial DNA, in splenic F4/80^+^ CD11b^+^ macrophages was significantly lower (Fig. 8a). Moreover, the IgM levels produced by spleen MNCs following CpG-ODN stimulation were significantly lower (Fig. 8b), and those produced by lipopolysaccharide (LPS)-stimulated spleen MNCs also tended to be lower (Fig. 8c). The proportions of B cells in the liver, kidney, and spleen did not differ between the two groups (Supplementary Fig. S2).

On the other hand, the IFN-γ levels produced by liver MNCs following stimulation with CpG-ODN were significantly higher in the α-GalCer group (Fig. 8d), suggesting that the function of NK cells was enhanced^24^. TLR-9 expression in hepatic F4/80^+^ CD11b^+^ macrophages did not differ between the two groups (data not shown).

## Discussion

To our knowledge, this is the first report to demonstrate that a repeated α-GalCer injection ameliorates podocyte injury, thereby inducing an anti-proteinuric effect. The number of WT-1 positive cells was significantly larger and the nephrin staining score was significantly higher in the α-GalCer group. Moreover, the preservation of podocyte foot processes was observed in the α-GalCer group. We assessed the proteinuria-free survival time in mice using two analysis methods; one showed a statistically significant difference, while another only suggested a tendency toward significance. Regarding this point, it has been reported that the log-rank test may show a tendency toward significant results^25^. Regardless, the daily levels of proteinuria in the α-GalCer group were significantly lower than those in the vehicle group.

In our study, the infiltration of F4/80-positive macrophages and CD3-positive T lymphocytes in the kidney was reduced, and the serum albumin/globulin ratio was higher in the α-GalCer group. A low albumin/globulin ratio reflects chronic inflammation^26^. Therefore, our data also suggested the resolution of inflammation following the repeated administration of α-GalCer.

It has been reported that NKT cells and B cells interact to generate Igs, including autoantibodies^27,28^, whereas inhibitory roles of NKT cells against autoreactive B cells have also been described^29^. In the present study, we showed another novel finding that the repeated administration of α-GalCer decreased glomerular immune complex deposits. Thus, the percentage of glomeruli containing immune complex deposits, as assessed by PAS staining, was significantly lower and the IgG deposition was significantly decreased in the α-GalCer group. These findings were also observed by electron microscopy. Moreover, the IgM levels produced by CpG-ODN-stimulated spleen MNCs were significantly lower. These data suggested the suppression of autoimmune responses mediated by a decrease in B cell function. The decreased TLR-9 expression of splenic macrophages in the α-GalCer group may also have been related to the improvement of kidney pathology, since TLR-9 has been implicated in the development of lupus nephritis^30^. Despite the alleviation of renal injuries, the administration of α-GalCer did not affect the levels of anti-dsDNA IgG Abs. However, in the present study, the levels of anti-dsDNA Abs did not correlate with the levels of proteinuria in BWF1 mice, suggesting that these autoantibodies are not pathogenetic markers. Similarly, a study using another SLE model demonstrated an improvement in skin lesions, without any changes in the levels of anti-dsDNA Abs^31^.

As previously reported^32^, a repeated α-GalCer injection induced anergy in NKT cells. In addition, the proportions of NKT cells in the liver, kidney, and spleen were significantly reduced in the α-GalCer group. In contrast, the IFN-γ levels remained constant following stimulation with ConA and anti-CD3 Abs, showing that the anergy observed in NKT cells was specific to α-GalCer.

On the other hand, the IL-4 levels produced by ConA and anti-CD3-stimulated liver MNCs were significantly reduced in the α-GalCer group, suggesting the suppression of Th2 immune responses. A predominant Th2 cytokine response in human membranous lupus nephritis has been reported^33^, and important roles for the Th2 immune responses in the pathogenesis of membranous lupus nephritis have been suggested^34,35^. Therefore, the present study strongly supported that the repeated administration of α-GalCer ameliorated the progression of lupus nephritis through the suppression of Th2 immune responses. However, it has also been reported that an α-GalCer injection modulates immune responses towards a Th2 phenotype in B6 mice^36^ and that Th2-biased immune responses induced by the administration of α-GalCer^17,18^ or its derivative^37^ prevented diabetes in NOD mice. Thus, the effects of α-GalCer could vary depending on the disease model.

Yang *et al*. demonstrated that a brief treatment with α-GalCer in 7-week-old BWF1 mice suppressed lupus nephritis^20^. On the other hand, Zeng *et al*. reported contradictory results, namely that multiple injections of α-GalCer in 20-week-old BWF1 mice exacerbated lupus nephritis^19^. Regarding this point, a complex role for NKT cells in SLE, that is, a potential protective role before the onset of the disease and a potential pathogenic role after the establishment of the disease has been proposed^16^. Moreover, the age of the mice and the α-GalCer dose or administration interval may have influenced these conflicting results. However, we considered that the administration of α-GalCer to young mice was clinically unsuitable, because current international guidelines do not recommend the treatment of patients with mild lupus nephritis using immunosuppressive therapies^38^. We, therefore, began the α-GalCer treatment at 24 weeks of age, when mice may already develop mild renal disease.

Although some experimental agents have been used in SLE models with favorable outcomes^22,39^, in the present study we did not use such agents. Instead, we administered α-GalCer, a synthetic but specific ligand of NKT cells, to investigate the role of these cells in lupus nephritis. Definitive natural ligands of NKT cells have not been so far identified. However, considering that α-GalCer is a glycolipid antigen and that some microbes may be antigenic candidates for NKT cells^40,41^, we think that NKT cells are involved in lupus nephritis via the glycolipid metabolism or in response to microbes.

We have recently demonstrated that the function of NKT cells was enhanced in the absence of NK cells (Uchida *et al*. in submission). In the present study, we have demonstrated that IFN-γ levels produced by CpG-ODN-stimulated liver MNCs were significantly higher in the α-GalCer group. Moreover, because we have previously reported that NK cells produced IFN-γ after CpG-ODN stimulation^24^, we proposed a compensatory augmentation in the function of NK cells. Although the precise mechanisms remain to be elucidated, complementary roles may be shared between NKT and NK cells to avoid immunosuppressive states.

Our study has several limitations. It has been reported that BWF1 mice are superior to other SLE models in that they resemble the human disease, and therefore, are most commonly used^42^. Indeed, these mice spontaneously develop lupus nephritis-like renal lesions. However, the renal disease observed in these mice does not fully recapitulate that in humans; for example, hematuria was not observed in BWF1 mice despite proliferative glomerulonephritis. The role of NKT cells in the progression of human lupus nephritis also remains to be resolved using α-GalCer. This is partly because human NKT cells, which express T cell receptors encoded by the Vα24Jα18 and Vβ11 genes and respond to α-GalCer, are rare in the liver as well as other organs. In addition, decreased levels of human CD56^+^ T cells, which are not activated by α-GalCer but have been proposed as a functional counterpart for mouse NKT cells^8,43,44^, might be associated with high levels of serum IgG and anti-dsDNA Abs in patients with SLE^14^, suggesting that CD56^+^ T cells may ameliorate SLE. Therefore, it should be carefully evaluated in the future whether human NKT cells or CD56^+^ T cells are really involved in lupus nephritis.

In conclusion, the repeated administration of α-GalCer decreased the number of NKT cells and suppressed Th2 immune responses in these cells. This led to the suppression of B cell function and the autoimmune response, and ameliorated proteinuria and kidney pathology in experimental lupus nephritis. Therefore, we propose that NKT cells collaborate with B cells and play significant roles in the induction of lupus nephritis through their Th2 immune responses. Thus, it is important to focus on NKT cells to further investigate the precise pathogenesis of lupus nephritis.

## Methods

### Mice and study protocols

Female BWF1 and B6 mice were obtained from Japan SLC, Inc. (Shizuoka, Japan), and were provided with water and standard chow ad libitum.

The baseline urinary protein excretion of BWF1 mice was measured at 20 weeks of age, and the mice were assigned to either the α-GalCer group (*n* = 8), which was intraperitoneally injected with 2 μg/body of α-GalCer (Funakoshi, Tokyo, Japan) once a week for 4 weeks from 24 weeks of age, or the vehicle group (*n* = 10). The baseline urinary protein excretion was balanced between the two groups and amounted to less than 1 mg/day. The urinary protein excretion was thereafter measured periodically, and was considered to be positive when greater than 1 mg/day.

B6 mice were intraperitoneally injected with 2 μg/body of α-GalCer once a week for 4 weeks from 24 weeks of age.

Mice were sacrificed under deep anesthesia at the end of the experiment (8 weeks after the last α-GalCer injection). All animal experiments were conducted in accordance with the National Defense Medical College guidelines for the care and use of laboratory animals in research. The study protocol (no. 15087) was approved by the Animal Ethics Committee of the National Defense Medical College.

### Assessment of renal histopathology

Sections of formalin-fixed paraffin-embedded renal tissues were subjected to PAS staining, and 50 glomeruli were evaluated to assess glomeruli with immune complex deposits consisting of wire loop lesions and hyaline thrombi, sclerotic glomeruli including both globally and segmentally sclerotic glomeruli, and glomeruli with crescents.

### Immunoperoxidase staining

Indirect immunoperoxidase staining for F4/80, CD3, or WT-1 was performed using rat anti-mouse F4/80 (Serotec, Oxford, UK), rabbit anti-human CD3 (Agilent, Santa Clara, CA), or rabbit anti-mouse WT-1 Abs (Santa Cruz Biotechnology Inc., Santa Cruz, CA), respectively.

Images of five non-overlapping areas from each section stained for F4/80 or CD3 were obtained with a digital camera at a magnification of 200×, and the percentages of the areas positive for each staining were measured using image analysis software (LuminaVision ver. 2.04, Mitani Corporation, Tokyo, Japan) and averaged. For WT-1 staining, 20 glomeruli were assessed, and the numbers of WT-1-positive cells per glomerulus were counted and averaged.

### Real-time reverse transcription-polymerase chain reaction (RT-PCR)

Extraction of total RNA, reverse transcription into complementary DNA, and the subsequent real-time PCR were all performed as previously described^45^. We used primer/probe sets of the TaqMan Gene Expression Assays for mouse CD11c, CD138, and glyceraldehyde 3-phosphate dehydrogenase (*GAPDH*), all obtained from Thermo Fisher Scientific (Waltham, MA). The relative amount of mRNA was calculated using the comparative Ct (∆∆Ct) method. All amplification products were normalized against *GAPDH* mRNA, which was amplified in the same reaction as an internal control.

### IF staining

Direct IF staining for IgG was performed using FITC-labeled anti-IgG Abs (MP Biomedicals, Santa Ana, CA), and indirect IF staining for nephrin was performed using anti-guinea pig nephrin Abs (Progen, Heidelberg, Germany).

The MFI for IgG staining was calculated by measuring and averaging the IF intensities of 20 randomly selected glomeruli under the same conditions using the image analysis software. For nephrin staining, 20 randomly selected glomeruli were graded semi-quantitatively as follows: 0, severely decreased staining; 1, mildly decreased staining; 2, intense staining. The nephrin IF score was then calculated using the formula: (0 × N0 + 1 × N1 + 2 × N2)/(N0 + N1 + N2), where N is the number of the glomeruli for each staining grade.

### Electron microscopy

Renal cortical tissue fragments were examined under a transmission electron microscope, essentially as previously described^46^.

### Cytokine measurements and biochemical parameters

The cytokine and IgM levels in the sera or culture supernatants, and the levels of serum IgG anti-dsDNA Abs were measured using ELISA kits (IL-4 and IFN-γ: BD Biosciences, San Diego, CA; IgM: Bethyl Laboratories, Inc., Montgomery, TX; IgG anti-dsDNA Abs: Shibayagi Co. Ltd., Gunma, Japan). BUN levels and serum albumin, total protein, and alanine aminotransferase levels were measured using a DRICHEM 3000 V system (Fuji Medical System, Tokyo, Japan). Urinary protein excretion was measured by a clinical laboratory testing company (SRL, Tokyo, Japan).

### Flow cytometry

Kidney, liver, and spleen specimens were filtered through a stainless-steel mesh and dissolved. Kidney MNCs were thereafter isolated using a Percoll density gradient (67% and 33%) centrifugation. Liver MNCs were isolated without collagenase, essentially as previously described^10^.

For the identification of whole NKT or iNKT cells, the MNCs were stained with anti-TCR αβ Ab (H57-597, eBioscience, San Diego, CA) and either anti-NK1.1 Ab (PK136, eBioscience) or the α-GalCer-loaded CD1d tetramer (MBL, Nagoya, Japan), respectively. Macrophages were identified by staining MNCs with anti-F4/80 (BM8, eBioscience) and anti-CD11b Abs (M1/70, eBioscience), and their expression of TLR-9 was examined using anti-TLR-9 Ab (J15A7, BD Biosciences). B cells were identified by staining MNCs with anti-B220 Ab (RA3-6B2, eBioscience). Flow cytometry was performed on a Cytomics FC500 instrument (Beckman Coulter, Indianapolis, IN).

### Cell culture for MNCs

MNCs (5 × 10^5^ cells) of liver or splenic origin in 200 μL of medium were cultured with either α-GalCer (100 ng/mL), ConA (1 ng/mL, Vector Laboratories, Inc., Burlingame, CA), anti-CD3 Ab (10 μg/mL, eBioscience), CpG-ODN (20 μg/mL, Hycult Biotech, Uden, The Netherlands), or LPS from *E. coli* 0111:B4 (10 μg/mL, Sigma-Aldrich, St. Louis, MO) in 96-well flat-bottom plates. The culture supernatants were harvested 48 hours after seeding.

### Statistics

Data are expressed as the mean ± SEM. Differences between two experimental groups were assessed using the Student’s *t-*test, and those among more than three experimental groups were assessed by one-way ANOVA with Tukey’s HSD post hoc test. Differences in the proteinuria-free survival time and the survival time of the groups were analyzed using the log-rank test and the Cox proportional hazard model. *P*-values of < 0.05 were considered statistically significant. All statistical analyses were performed using the JMP software (version 11; SAS Institute Inc., Cary, NC).

### Data availability

All data generated or analyzed during this study are included in this published article (and its Supplementary Information files).

## Electronic supplementary material

Supplementary information
