# Supplementary material for: Repeated administration of alpha-galactosylceramide ameliorates experimental lupus nephritis in mice
Source: Sci Rep. 2018 May 29;8:8225. doi: 10.1038/s41598-018-26470-w (PMC5974230; doi:10.1038/s41598-018-26470-w)
Supplement: Supplementary file 1 — Supplementary information [file 41598_2018_26470_MOESM1_ESM.pdf]

SUPPLEMENTARY INFORMATION

Repeated administration of alpha-galactosylceramide ameliorates experimental lupus nephritis in mice

Takahiro Uchida, M.D.,[1, *] Hiroyuki Nakashima, M.D.,[2] Akira Yamagata, M.D.,[1] Seigo Ito, M.D.,[1] Takuya Ishikiriyama, M.D.,[2] Masahiro Nakashima, M.D.,[2] Shuhji Seki, M.D.,[2] Hiroo Kumagai, M.D.,[1] and Naoki Oshima, M.D.[1]

*Corresponding author. E-mail: DRI1905@ndmc.ac.jp

Supplementary Figure S1. Messenger RNA (mRNA) expression levels for the dendritic cell marker and plasma cell marker in the kidney

Supplementary Figure S2. The percentages of B cells in multiple organs

Supplementary Table S1. Crude hazard ratios (95% confidence interval) of repeated alpha-galactosylceramide administration for the incidence of proteinuria and survival rates

Supplementary Figure S1.

Messenger RNA (mRNA) expression levels for the dendritic cell marker and plasma cell marker in the kidney

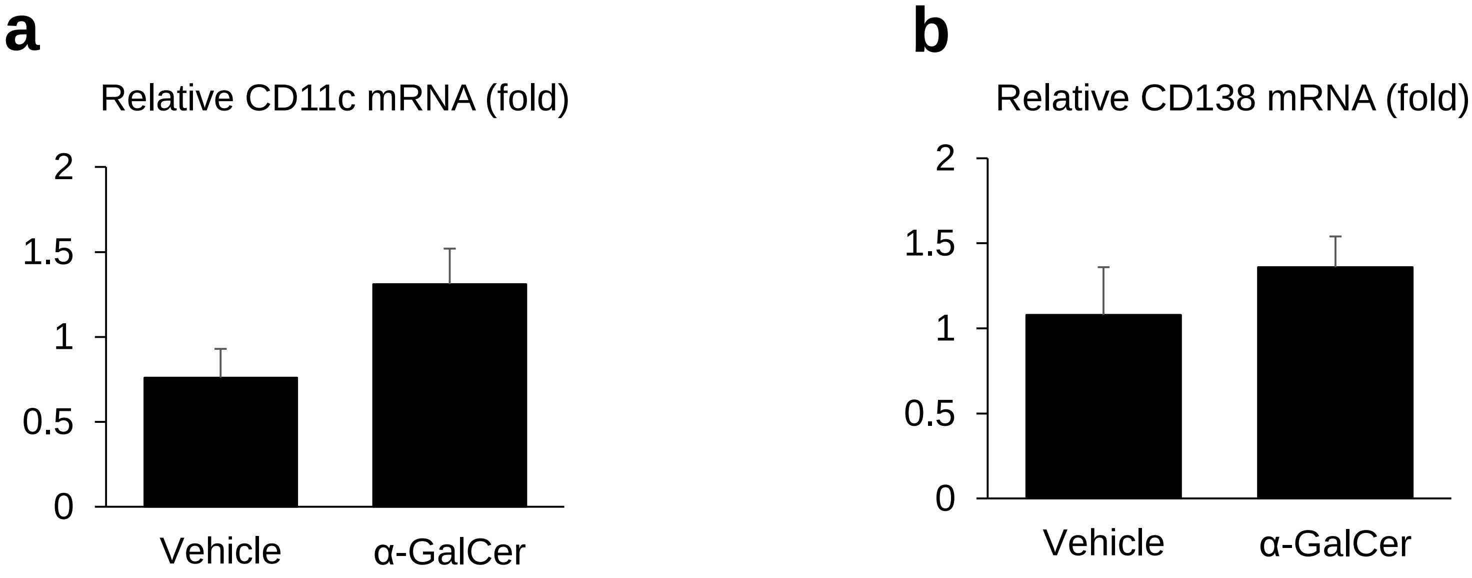

Relative renal mRNA expression levels of (**a**) CD11c and (**b**) CD138 in each group are shown (*n* = 4 in each group). Glyceraldehyde 3-phosphate dehydrogenase (*GAPDH*) mRNA was used as an internal control to adjust for total mRNA levels.

Supplementary Figure S2.
The percentages of B cells in multiple organs

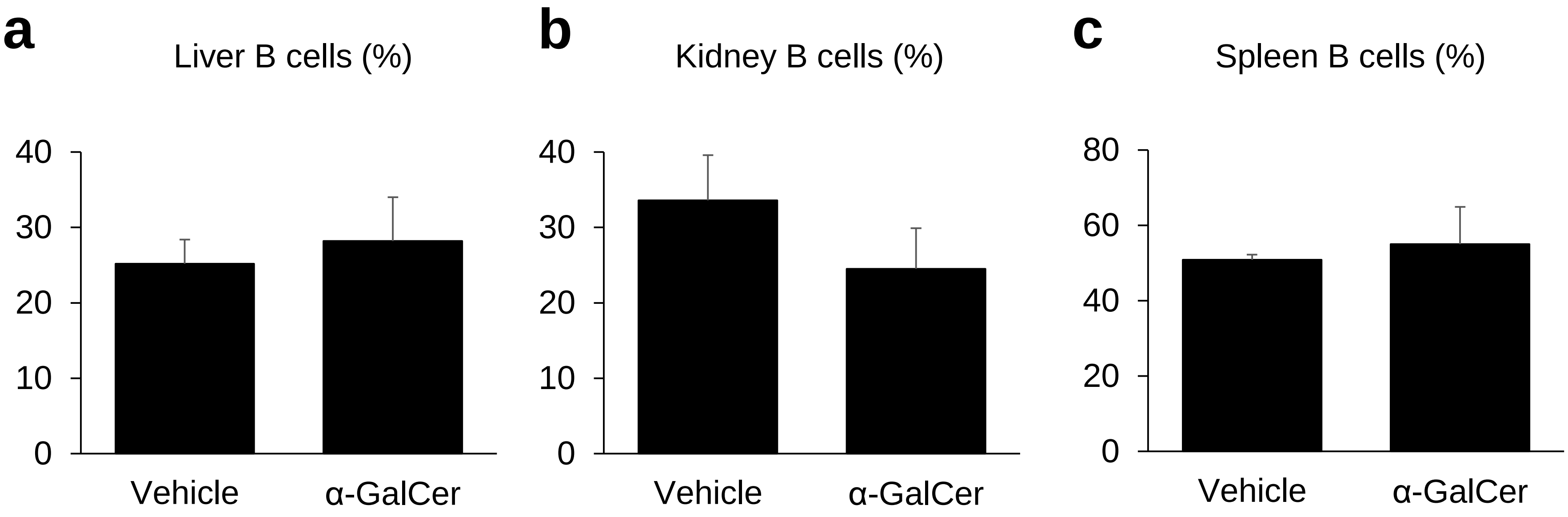

The ratio of B cells to total lymphocytes in the (**a**) liver, (**b**) kidney, and (**c**) spleen for each group are shown (*n* = 3-5 in each group). The lymphocytes were isolated 8 weeks after the last α-GalCer or vehicle injection.

Supplementary Table S1.

Crude hazard ratios (95% confidence interval) of repeated alpha-galactosylceramide administration for the incidence of proteinuria and survival rates

Characteristics	Hazard ratio	*P*-value
Incidence of proteinuria	0.38 (0.10–1.20)	0.10
Survival rates	0.35 (0.02–2.74)	0.33
